# High-speed identification of suspended carbon nanotubes using Raman spectroscopy and deep learning

**DOI:** 10.1038/s41378-022-00350-w

**Published:** 2022-02-10

**Authors:** Jian Zhang, Mickael L. Perrin, Luis Barba, Jan Overbeck, Seoho Jung, Brock Grassy, Aryan Agal, Rico Muff, Rolf Brönnimann, Miroslav Haluska, Cosmin Roman, Christofer Hierold, Martin Jaggi, Michel Calame

**Affiliations:** 1 Laboratory for Transport at Nanoscale Interfaces, Empa, Swiss Federal Laboratories for Materials Science and Technology, CH-8600 Dübendorf, Switzerland; 2 Machine Learning and Optimization Laboratory, School of Computer and Communication Sciences, EPFL, CH-1015 Lausanne, Switzerland; 3 Department of Physics and Swiss Nanoscience Institute, University of Basel, CH-4056 Basel, Switzerland; 4 Micro- and Nanosystems, Department of Mechanical and Process Engineering, ETH Zurich, CH-8092 Zurich, Switzerland

**Keywords:** Carbon nanotubes and fullerenes, Carbon nanotubes and fullerenes

## Abstract

The identification of nanomaterials with the properties required for energy-efficient electronic systems is usually a tedious human task. A workflow to rapidly localize and characterize nanomaterials at the various stages of their integration into large-scale fabrication processes is essential for quality control and, ultimately, their industrial adoption. In this work, we develop a high-throughput approach to rapidly identify suspended carbon nanotubes (CNTs) by using high-speed Raman imaging and deep learning analysis. Even for Raman spectra with extremely low signal-to-noise ratios (SNRs) of 0.9, we achieve a classification accuracy that exceeds 90%, while it reaches 98% for an SNR of 2.2. By applying a threshold on the output of the softmax layer of an optimized convolutional neural network (CNN), we further increase the accuracy of the classification. Moreover, we propose an optimized Raman scanning strategy to minimize the acquisition time while simultaneously identifying the position, amount, and metallicity of CNTs on each sample. Our approach can readily be extended to other types of nanomaterials and has the potential to be integrated into a production line to monitor the quality and properties of nanomaterials during fabrication.

## Introduction

Because of their nanoscale diameter and high carrier mobility, carbon nanotubes (CNTs) are prominent among a variety of nanoscale materials that have been considered for next-generation energy-efficient electronic systems^1^. For example, CNTs have been successfully used for chemical and physical sensing at unprecedented low power consumption in the range of μW per sensor function^2,3^. Recently, a beyond-silicon modern microprocessor was designed and fabricated entirely using CNT field-effect transistors (CNFETs)^4^. On the road to the commercialization of CNT-based electronics^1,5,6^, methods to rapidly characterize CNTs at various stages of large-scale fabrication processes are essential for quality control and for the industrial adoption of CNFET technology. In particular, Raman spectroscopy has the advantages of being a nondestructive, noncontact approach, providing chemical, and structural information with micrometer spatial resolution^7^. In addition, the position, width, and shape of the characteristic peaks in the Raman spectra are indicators of the molecular or crystalline structure, electronic properties, and the quality of the materials. In the case of CNTs, the so-called G band shape and resonant electronic Raman scattering are used to distinguish the type of CNT^8–10^. (see Supplementary information Fig. S1)

Due to the low efficiency of Raman scattering, subtle spectral signals are easily masked by background noise^8,11^. As a result, Raman imaging at high speed has been technically challenging because Raman spectroscopy usually requires relatively high power (in the milliwatt range) and long exposure times (hundreds of milliseconds to a few seconds) to reach the desired identification accuracies. This issue can be partially overcome by collecting Raman-scattered photons using a fast electron-multiplying charge-coupled device (EMCCD), improving the signal-to-noise ratio (SNR) at scanning speeds below 1 millisecond per spectrum^12,13^. Nevertheless, at such low integration times, achieving a reliable distinction between different types of CNTs is challenging.

Machine-learning (ML) algorithms may offer a solution, as they have recently been used to solve complex recognition and classification problems, such as image^14^ and facial recognition^15,16^, as well as speech^17,18^ and text understanding^19,20^. Their underlying strength is their excellence at recognizing patterns, either by relying on previous experience (supervised ML) or without any a priori knowledge of the system itself (unsupervised ML)^21,22^. The number of tasks addressed by ML is growing every day, with many applications in image processing, e.g., digital pathology^23^, self-driving cars^24^, or image enhancement^15^. Of particular interest for classification purposes are artificial neural networks, networks of interconnected neurons that are trained on datasets of known classes to find patterns that distinguish these classes as well as possible. In recent years, deep neural networks consisting of multiple layers of various types (fully connected, convolutional, max pooling, dropout, etc.), have become the dominant tool in ML due to the remarkable results they have achieved^14–20^. Convolutional neural networks (CNNs), consisting mostly of convolutional layers, have been widely applied in computer vision for image classification or object recognition and are also playing an increasingly important role in the analysis of spectroscopic data, including Raman spectra^11,25^.

**Fig. 1:**
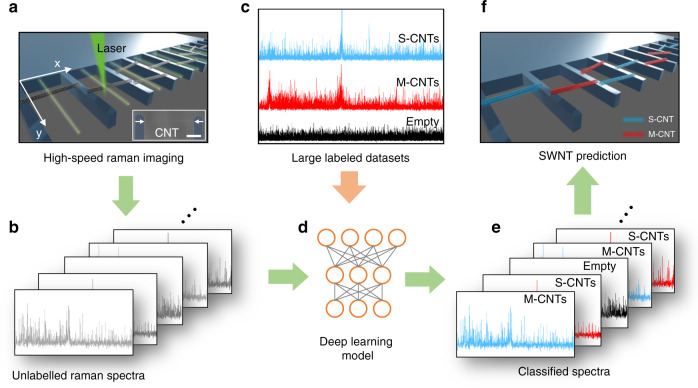
Schematic illustration of deep learning-based Raman spectra analysis for CNT identification. **a** Implementation of high-speed Raman imaging on a fork-like growth substrate. **b** Generation of unlabeled Raman spectra. **c** Large labeled datasets organized into three classes: S-CNTs, M-CNTs, and empty. **d** Deep learning model. **e** Classification of individual spectra using the model. **f** CNT identification.

Here, we develop a high-throughput approach to rapidly identify suspended CNTs based on the combination of deep learning, high-speed Raman spectroscopy, and an optimized scan strategy. We apply our approach to suspended CNTs that were grown on fork-like growth substrates that are optimized for a high-throughput dry transfer process. Such a process has been demonstrated to result in clean and CMOS-compatible CNT integration^26,27^ and has been used for ultralow-power gas sensors^2,3^ nanoresonators^28^, and quantum device applications^29,30^. The workflow of the identification is schematically depicted in Fig. 1. Individual CNTs are grown and suspended across SiO_2_/Si forks (see Fig. 1a). To achieve a high-throughput approach, we implement a line scan at each fork instead of using the time-consuming full-mapping method. We optimize the line-scan parameters to obtain sufficient spectral information while allowing for unambiguous localization of CNTs and classification into ‘M-CNTs’ ‘S-CNTs’, or ‘Empty’ (see methods). All the (unlabeled) Raman spectra obtained from the line scan (see Fig. 1b) are then classified individually using an ML model (see Fig. 1d), which is trained on a large collection of labeled Raman spectra (Fig. 1c). After the training was completed, new individual Raman spectra were classified using the trained machine-learning model (Fig. 1e). To improve the classification accuracy, we apply additional methods. First, we apply a threshold on the output of the softmax layer to reduce the number of false positives and the number of false negatives. Second, we developed an optimized scanning strategy that rescans areas of interest and identifies the position and amount of CNTs across each fork (Fig. 1f) with increased efficiency compared to line scans. We elaborate on each of these steps in the remainder of this article.

## Results and discussion

### Dataset generation

**Fig. 2:**
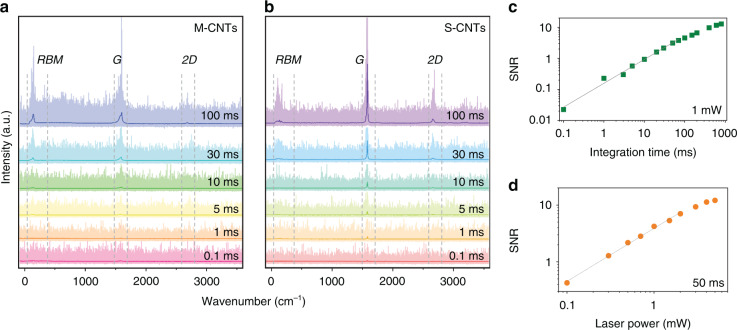
Raman analysis of CNTs. **a** Raman spectra of M-CNTs with various integration times at fixed power (1 mW). At each setting, the average of 1250 spectra from 32 M-CNTs are shown in bold and overlaid on these single spectra. Spectra are colored according to integration time. **b** Raman spectra from 30 S-CNTs, with the same data size and plotting formats as in **a**. **c** SNR versus integration time at fixed power (1 mW). **d** SNR versus laser power at a fixed integration time (50 ms).

To minimize the scan time of each fork, the Raman spectra need to be acquired using an integration time as short as possible. Short integration times lead to low SNR and a reduction in the classification accuracy of the machine-learning model. Shorter integration times can be compensated by higher laser intensities up to the point where damage to the CNTs occurs. To investigate this trade-off between speed and accuracy and to identify the optimum settings, we built a range of datasets consisting of Raman spectra acquired using different integration times (ranging from 1 to 600 ms at 1 mW power) and different laser powers (ranging from 0.1 to 3 mW for an integration time of 50 ms) on suspended CNT samples. An ML model was trained on the training dataset and tested on a separately acquired validation dataset, acquired in a similar fashion as the training dataset. To train the ML model, we constructed a large-scale labeled dataset consisting of 62,130 spectra at 19 Raman settings with different integration times and laser powers, acquired on 21 trenches containing CNTs with Raman characteristics of metallic CNTs (M-CNTs), 20 trenches containing CNTs with Raman characteristics that do not correspond to typical metallic CNTs (S-CNTs), and 20 trenches where no CNTs were found. In addition, we separately constructed labeled datasets for the validation step consisting of 48,887 spectra acquired on 10 trenches containing S-CNTs, 11 trenches containing M-CNTs, and 15 trenches where no CNTs were present. Figure 2a and b shows the average spectra acquired at various integration times for S-CNTs and M-CNTs. The spectra acquired at various laser powers for S-CNTs and M-CNTs are shown in Supplementary Information Fig. S2. The plots also display the overall envelope of all spectra (1250 spectra for each setting) as a shaded background. Here, the overall spread is significantly larger than for an averaged spectrum, highlighting the large spectrum-to-spectrum variability. Additionally, for the lower integration times, the SNR is drastically reduced. We quantified the SNR, and we show its dependence on the integration times and laser power in Fig. 2c and d. As expected, in both plots, the SNR scales linearly with integration times and laser power. We note that an SNR below 1 implies that the signal cannot be distinguished from noise, as is the case for all integration times below 10 ms at 1 mW and 0.1 mW with 50 ms.

### Training and validation of the neural network

**Fig. 3:**
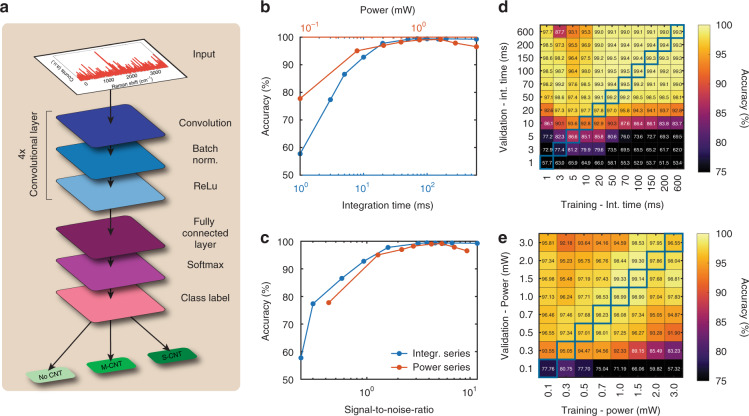
Training and validation of the deep learning model. **a** Architecture of the convolutional neural network. **b, c** Accuracy of the model while varying the integration times used for training and validation. **d, e** Accuracy of the model while varying the laser power used for training and validation.

The neural network architecture used for classification (see Fig. 3a) was obtained using a gridsearch through various neural network architectures (see Supplementary information: Part 2). The optimal network was found to be a convolutional neural network (CNN) (Fig. 3a), with 4 convolutional layers followed by fully connected layers, a softmax layer, and finally a cross-entropy layer for defining the loss function. More details about the network architecture are provided in the Methods section. To explore the characteristic bands extracted by the model, we plotted the activation values for the first convolutional layer in the plots below for a randomly selected spectrum acquired on semiconducting and metallic CNTs, as shown in Supplementary Information Fig. S3. The results are in agreement with the Raman-active CNT peaks and corroborate the fact that the network is activated by the characteristic spectral features.

The classification accuracies obtained for datasets with different integration times and laser powers are presented in Fig. 3b. The accuracy is ~60% for the shortest integration time (0.1 ms), increases to >90% at 10 ms and reaches >98% for >30 ms. The accuracy for the power dependence starts at 78% for 0.1 mW and reaches a maximum of 99% at 1 mW, after which it slightly decreases to 96%. This decrease is likely caused by heating or damage from the intense laser irradiation of the CNTs^31,32^. Figure 3c presents the same accuracies, plotted against the SNRs obtained in Fig. 2c and d.

Furthermore, we explore the generalization of a model trained on specific Raman settings (power and integration time) and tested on data acquired with different settings. This analysis is relevant for investigating the accuracy of the model in conditions in which the test data do not match the same acquisition process as the training data. This could, for instance, be due to the drift of the experimental parameters or other temporary limitations arising during the characterization process. For completeness, for each integration time at which the model is trained, the accuracy for all integration times is obtained, yielding a matrix of all accuracies (see Fig. 3d), with the parameters used for training on the horizontal axis and those for validation on the vertical axis. The same procedure is repeated for power dependence (see Fig. 3e). We note that the highlighted accuracies on the diagonal of the matrix correspond to identical training and validation parameters and are identical to those presented in Fig. 3b. We find that the model is remarkably robust against variation. Additionally, models trained on low SNR data perform well on higher SNR data, but not the opposite. In fact, models trained on high SNR datasets perform poorly on low SNR datasets. For varying integration times, accuracies of >90% are reached when testing on 20 ms data, independent of the testing parameters, while for testing and training times >50 ms, all combinations yield >98%. We note several outliers in the plot (such as training 3 ms and validation 600 ms, which we attribute to outliers in the dataset). Nevertheless, the overall trend is robust. For the power dependence, we find that the accuracy is the highest close to the diagonal and decreases when moving away from it. Again, we find that models trained on low SNR datasets perform well on high SNR datasets, but not vice versa.

### Softmax thresholding

**Fig. 4:**
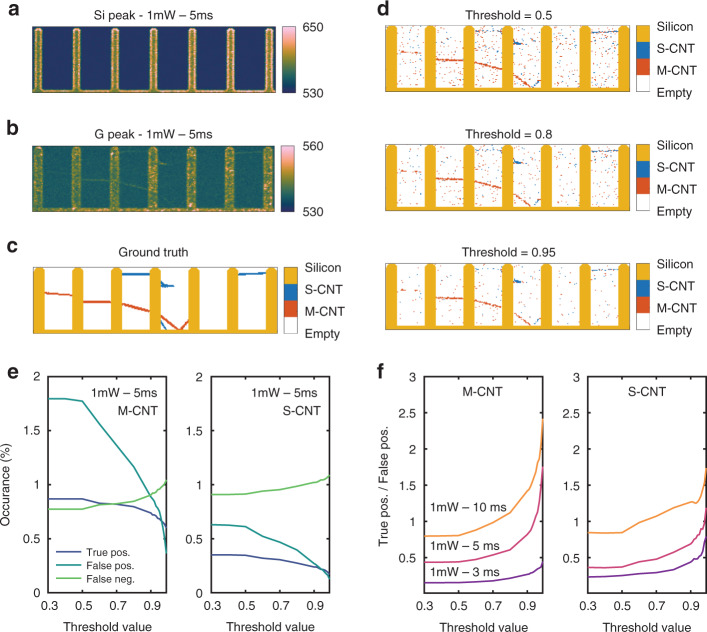
Classification of a Raman map for varying threshold values. **a** Raman intensity map of the Silicon peak. **b** Raman intensity map of the G-peak. **c** Ground truth map. **d** Map of the predicted classes for varying softmax threshold values. **e** Occurrence of true positives, false positives, and false negatives for varying softmax threshold values for M-CNTs and S-CNTs. **f** Ratio of false-positive/true positives for varying softmax threshold values for M-CNTs and S-CNTs.

With the deep learning model being trained and its accuracy assessed, we proceed with its application to a map acquired on a series of forks. Figure 4a and b shows the intensities of the silicon (520 cm^−1^) and G-peak (~1580 cm^−1^), respectively, obtained from a Raman map acquired at a low integration time of 5 ms and a laser power of 1 mW focused on CNTs with an objective magnification of ×50 and NA of 0.55. Figure 4c presents the ground truth established using Raman spectra acquired with integration times of 800 ms and 1 mW. We note that the silicon labels do not originate from the deep learning model but are obtained by applying a thresholding function to the intensity of the silicon peak and that there may be CNTs that are undetectable with the laser wavelength used. To increase the confidence of the classification by the neural network for fast map scans, we apply a threshold to the output of the softmax layer. This means that the S-CNT and M-CNT classes are only used if the confidence of the network for these classes exceeds a predefined threshold value. If this is not the case, the pixel in question is classified as Empty. Figure 4d shows the fast map with each pixel classified for three selected threshold values. The plot for a threshold of 0.5 qualitatively reproduces the ground truth, albeit with a large amount of noise (randomly distributed pixels that are misclassified). For an increasing threshold, the noise gradually decreases, as does—to a lesser extent—the number of correctly classified pixels. To quantify the efficacy of the thresholding approach, Fig. 4e shows the number of true positives (TP), false positives (FP), and false negatives (FN) for M-CNTs and S-CNTs as a function of the threshold value. The threshold ensures that only the S/M pixels with a high classification fidelity (value at softmax layer) remain in the S/M class. Increasing this threshold therefore drastically reduces the number of FPs, which usually have a relatively low activation at the softmax layer, as well as some of the TPs. In addition, the FN, i.e., the pixels that should have been S/M but are wrongly classified will increase as the criteria to be in the correct class become more stringent with an increasing threshold.

Importantly, the ratio between TP and FP is shown in Fig. 4f for M-CNTs and S-CNTs. The threshold value increases from 0.3 up to 0.99, and by doing so, the true positive to false-positive ratio improves by a factor of approximately 3 when classifying at an integration time of 5 ms. As a comparison, we also included the same curve for integration times of 3 ms and 10 ms in the plot. For higher integration times, the classification accuracy measured by the TP-to-FP ratio increases.

### CNT identification

**Fig. 5:**
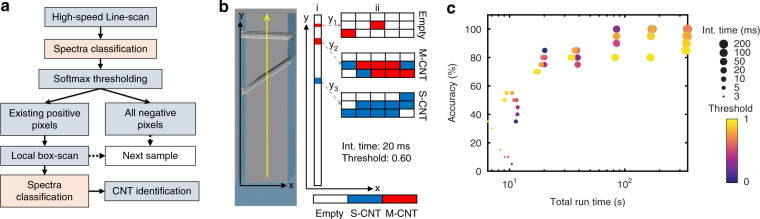
CNT identification with a ‘box-scan’ approach. **a** Workflow of CNT identification. **b** An example of CNT prediction: (i) high-speed line scan (20 ms/pixel) across a trench and spectral classification: (ii) box-scan (20 ms/pixel) with 3 × 5 pixels on the spots of positive pixels in (i) and spectral classification. After analysis, one M-CNT at position y_2_ and one S-CNT at position y_3_ are identified. **c** Accuracy versus total run time for the scanning of 20 trenches while varying the integration time (marker size) and threshold (color). The same integration time is used for the Line scan and BoxScan.

Although applying a threshold to the softmax output can effectively increase the confidence of the classification, it is still challenging to accurately predict the number and type of CNTs in each trench. Moreover, a map scan with resolution-limited pixel spacing results in prohibitively long measurement times, even for the shortest single-pixel integration times. To address these issues, we propose a proof-of-concept workflow employing an initial line scan to detect CNTs in a trench, followed by an optimized ‘box scan’ approach that rescans areas of interest for CNT identification (Fig. 5a). From the initial high-speed line scan (25 μm with 75 pixels), the spectra are fed into the network model for classification. After applying a threshold to the output of the softmax layer, each pixel in the line map is classified as either negative (empty) or positive (S-CNT or M-CNT). If all the pixels are classified as negative, a high-speed line scan will take place in the next trench. Otherwise, the laser will move back to the positions of the positive pixels and implement a larger area box-scan (3 × 5 pixels) with the same integration time as in the line scan. Then, these box maps (15 spectra/box) will be input into the CNN for classification. Based on the classification results of the spectra in each box map, the number and type of CNTs in each trench are estimated using two simple criteria: (1) In each box, if the number of positive N_P_s > 2, one CNT is identified; otherwise, no CNT is assumed to be present in this box. (2) In a CNT box, if the number of M-CNT-predicted pixels (*N*_M_) is more than those of S-CNT-predicted pixels (*N*_S_), N_M_ > N_S_, this CNT is assumed to be an M-CNT, or otherwise a S-CNT. These criteria are obtained with the highest prediction accuracies on the samples in Fig. 5c.

Figure 5b shows an example of the CNT prediction. After a high-speed line scan across a trench, spectra are classified using the network model. After classification and softmax thresholding (threshold = 0.6), 5 pixels at 3 spots, y_1_, y_2,_ and y_3_, are classified as positives. Then, larger area box scans with 3 × 5 pixels are performed on these three spots in (i), followed by the classification for each box (ii). After analysis, one M-CNT at position y_1_ and one S-CNT at position y_2_ are identified. To assess the usefulness of this approach, Fig. 5c shows the identification accuracy and total run time (laser exposure time) of the identification process for 20 trenches (containing 15 CNTs) while varying the integration time and threshold. The total run time is the sum of the integration time from the line scan and (conditional) box-scan and therefore depends on both the integration time and threshold of the softmax layer. The overall accuracy increases sharply with integration time from 3 to 10 ms and then slowly levels off. As a trade-off, the total run time increases with an increase in integration time. For a set integration time, there is an intricate dependence on the threshold value of the softmax layer: for very short integration times, where the ratio of true pos./false pos. classification is low, a higher threshold value will increase both accuracy and reduce measurement time because time-consuming box scans returning empty results are avoided. In contrast, for long integration times, a higher threshold will sacrifice the accuracy due to the removal of a fraction of true positives (Fig. 4e) but will still reduce the run time. Consequently, the targeted identification accuracy is a crucial parameter for the choice of an optimal value for both integration time and the threshold value in the softmax layer. It is important to note that from an application point of view, this optimal accuracy target will depend on a potentially complicated cost function taking into account a variety of parameters, such as the dynamics of the mechanical positioning/scanning system, speed of data communication between acquisition software and CNN, resulting hardware-dependent total run time, and the cost function of misidentified CNTs for downstream processes. These parameters can vary widely depending on the application and whether the process is carried out on a research setup (such as the WITec Alpha 300 R employed here) or a dedicated production setup running the CNN on dedicated hardware in the Raman control loop. Additionally, other techniques, such as compressive Raman spectroscopy^33,34^ and multifocal Raman spectroscopy^35,36^, may give rise to a higher scanning rate and higher SNR improvement.

## Conclusion

In conclusion, we implemented a high-throughput high-accuracy approach to identifying suspended CNTs using a Raman spectroscopy line-scan method and deep learning classification method. With this approach, we significantly increased Raman scanning rates compared to large-scale spectroscopic mapping with human-based spectral postprocessing. Reducing the exposure time to only a few milliseconds minimizes laser-induced sample damage, while an appropriately trained CNN permits the quantitative identification of CNTs on the growth substrate, providing information about their number, position, and physical properties. We expect our high-throughput approach to be applicable to other emerging nanoscales. It allows the integration of a fast, reliable, quantitative, and spatially resolved nanomaterial identification process within an assembly workflow for the integration of nanoscale materials in technological applications.

## Materials and methods

### Sample preparation

The suspended CNT samples were prepared by the same methods as described in a recent work^2,26^. The fork-like substrates were fabricated by using standard photolithography and wet etching on a SiO_2_/Si wafer. After the deposition of iron-loaded ferritin particles (as catalyst precursors) on the fork structures, suspended CNTs were grown by chemical vapor deposition (CVD) in a CH_4_/H_2_ atmosphere at 825 °C for 30 min. After growth, ~50% of the forks were successfully bridged with 1–3 CNTs, while the rest of the forks remained empty.

### Raman measurements

We measured Raman spectra across the fork-like substrate using a confocal Raman microscope (WiTec, alpha300 R). A 532 nm laser was used with a 600 1/mm grating to generate spectra. Wavenumber and pinhole calibration was performed using a standard silicon sample. A long focal length ZEISS 50× 0.55 An NA objective lens was used, yielding a diffraction-limited spot size of ~1.2 μm in diameter. To collect a large dataset for network training, we first took a map scan on the silicon finger area with a pixel density of 3 pixels per μm^2^ (in both the x- and y-directions) using the settings of 1 mW power and 20 ms integration time. Then, we set a narrow window region (0.1 × 5 μm^2^) on top of the CNR area and performed map scans with various integration times and powers. The Raman settings for the network training dataset are (power/integration time): 0.1 mW/50 ms, 0.3 mW/50 ms, 0.5 mW/50 ms, 0.7 mW/50 ms, 1 mW/50 ms, 1.5 mW/50 ms, 2 mW/50 ms, 3 mW/50 ms, and 1 mW/1 ms, 1 mW/3 ms, 1 mW/5 ms, 1 mW/10 ms, 1 mW/20 ms, 1 mW/50 ms, 1 mW/70 ms, 1 mW/100 ms, 1 mW/150 ms, 1 mW/200 ms, 1 mW/600 ms. We collected the dataset from an empty sample using the same Raman settings as for the S- and M-CNTs. Finally, we obtained a total of 62,130 labeled training spectra acquired on 20 trenches containing S-CNTs, 21 trenches containing M-CNTs, and 20 trenches where no CNTs were present. In addition, we obtained separately labeled datasets for validation consisting of 48,887 spectra acquired on 10 trenches containing S-CNTs, 11 trenches containing M-CNTs, and 15 trenches where no CNTs were present. In the high-speed line-scan experiment, a Raman map (0.5 μm in the x-direction and 20 μm in the y-direction) with 2 lines and 75 pixels per line was scanned in the middle position of each trench. Please note that we needed to set at least 2 lines for each map scan in the WiTec Raman microscope, but only one line was taken for the analysis.

Our Raman system is equipped with three objectives: 10 × 0.25 NA, 50 × 0.55 NA, and 100 ×0.9. The 10 × 0.25 NA objective has a low spatial resolution since its diffraction-limited spot size is as large as ~2.6 μm in diameter. Given the length of the finger of the growth substrate of 20 μm, with this spot size, it would be challenging to distinguish two CNTs that are grown in the same trench within 3 μm from each other. On the other hand, the 100 × 0.9NA objective has a much higher spatial resolution of ~0.72 μm but a very shallow depth of focus (FWHM ~ 0.81 μm); it is therefore not suitable for this application as the CNTs are not perfectly straight and may have some bending. We find that the 50 × 0.55 NA objective, with an intermediate diffraction-limited spot size of ~1.2 μm and FWHM of the depth of focus being ~3.2 μm, is a good trade-off.

### SNR calculation

The SNRs of the CNT spectra for each Raman setting (see Supplementary information Fig. S4) is determined from:

1 $${\mathrm{SNR}} = \frac{{{\mathrm{Mean}}\left( G \right) - {\mathrm{Mean(backgroud)}}}}{{\Delta ({\mathrm{background}})}},$$

where Mean*(G)* is the average intensity of the G band region (1540–1605 cm^−1^) and Mean(background) is the average intensity at a region (2000–2200 cm^−1^) where no bands of CNTs occur. Δ(background) is the standard variation calculated from the same spectrum in the region 2000–2200 cm^−1^. For our datasets, the SNR variance for each Raman setting is quite high because of the spatial position variance of the CNTs with respect to the focusing position of the laser beam. When intradataset variance is high, a large number of spectra per Raman setting may help to better represent the full data distribution and lead to higher predictive performance.

### CNN architecture

The Raman spectra (dimensions 1600 × 1) are fed to a neural network in which the layout has been optimized using gridsearch through a various residual neural network (ResNet) and convolutional neural network (CNN) architectures with various hyperparameters (see Supplementary information: Part 2). We found that the optimized network is a regular CNN consisting of 4 blocks, each consisting of a convolutional layer, a batch normalization layer, and a ReLU activation layer. The convolutional layer contains 192 filters of size 13 × 1 that have a stride of 2 and a padding of 6 such that the size of the input is reduced by half (800, 400, 200, and 100) after each convolutional layer. The network is completed with a fully connected layer in which the number of neurons matches the number of classes (3), a softmax activation layer and a cross-entropy layer to define the loss function. Finally, we note that for all Raman spectra, cosmic ray removal is performed individually, as well as a standardization step (mean = 0, standard deviation = 1).

## Supplementary information

Supporting Information

## Data Availability

The data are available from the corresponding authors upon reasonable request.

## Code availability

The codes are available from the corresponding authors upon reasonable request.
