# Supplementary material for: High-speed identification of suspended carbon nanotubes using Raman spectroscopy and deep learning
Source: Microsyst Nanoeng. 2022 Feb 10;8:19. doi: 10.1038/s41378-022-00350-w (PMC8828464; doi:10.1038/s41378-022-00350-w)
Supplement: Supplementary file 1 — Supporting Information [file 41378_2022_350_MOESM1_ESM.docx]

Supplementary information for

High-speed identification of suspended carbon nanotubes using Raman spectroscopy and deep learning

Jian Zhang^1,§^, Mickael L. Perrin^1,§ ,*^, Luis Barba^2^, Jan Overbeck^1,4^, Seoho Jung^3^, Brock Grassy^2^, Aryan Agal^2^, Rico Muff^1^, Rolf Brönnimann^1^, Miroslav Haluska^3^, Cosmin Roman^3^, Christofer Hierold^3^, Martin Jaggi^2^, Michel Calame^1,4,*^

^1^Empa, Swiss Federal Laboratories for Materials Science and Technology, CH-8600 Dübendorf, Switzerland;

^2^Machine Learning and Optimization Laboratory, School of Computer and Communication Sciences, EPFL, CH-1015 Lausanne, Switzerland

^3^Micro- and Nanosystems, Department of Mechanical and Process Engineering, ETH Zurich, CH-8092 Zurich, Switzerland

^4^Department of Physics and Swiss Nanoscience Institute, University of Basel, CH-4056 Basel, Switzerland;

^§^J. Zhang and M. L. Perrin contributed equally.

^*^Email: mickael.perrin@empa.ch; [michel.calame@empa.ch](mailto:michel.calame@empa.ch)

**Part 1: Additional data on training datasets**

**
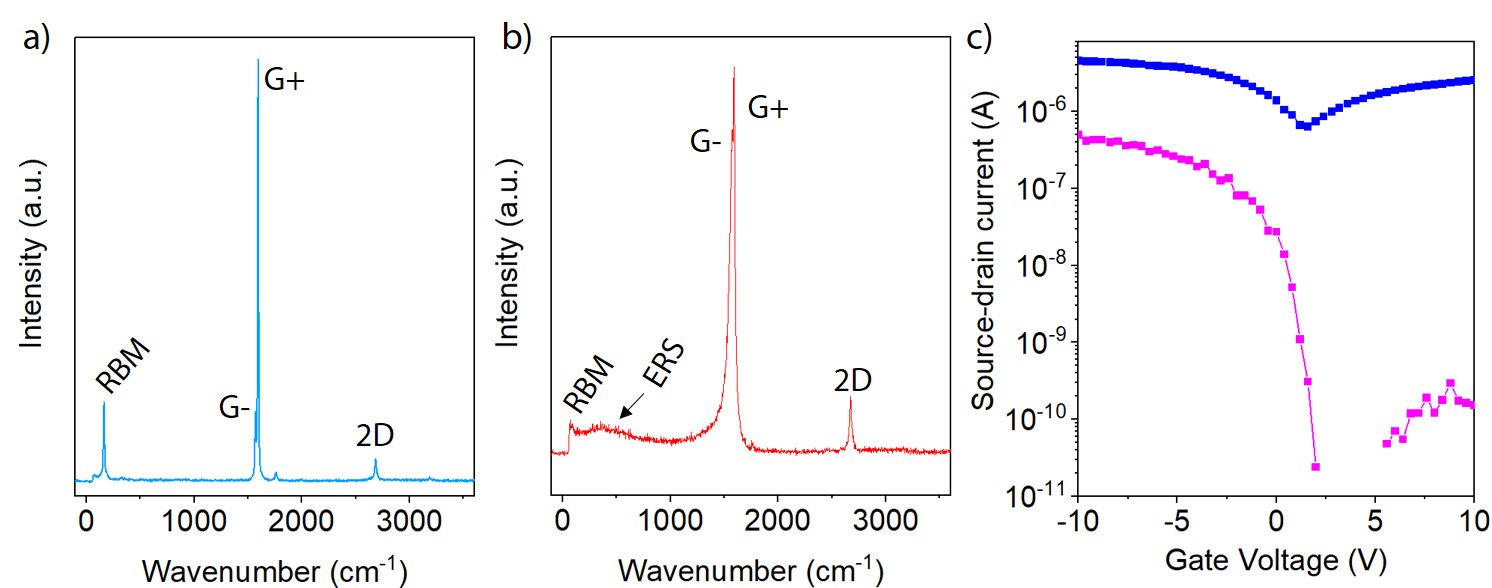
**

**Fig. S1** Raman spectra typical for S-CNT (a) and M-CNT (b).^1^ The spectra were acquired under 532 nm laser excitation with 1 mW and 20s. For the CNT, the G band (∼1600 cm^-1^) splits into two peaks: G+ and G-, whose splitting in frequency (ω_G+_ - ω_G-_) shows a different dependence on the CNT diameter between metallic and semiconducting CNTs (M-CNTs and S-CNTs).^1,2^ In the case of metallic CNTs, the G- peaks soften, broadening with an asymmetric spectral lineshape. Meanwhile, the resonant electronic Raman scattering features (ERS), which are resonantly enhanced at the corresponding excitonic transition energies, can be observed for metallic CNTs.^3,4^ (c) Representative transfer characteristics (source-drain current *vs.* gate voltage) of a semiconducting (bottom) and a metallic (top) device. The transferred CNT devices were fabricated and measured by the same methods as described in a recent work.^5^


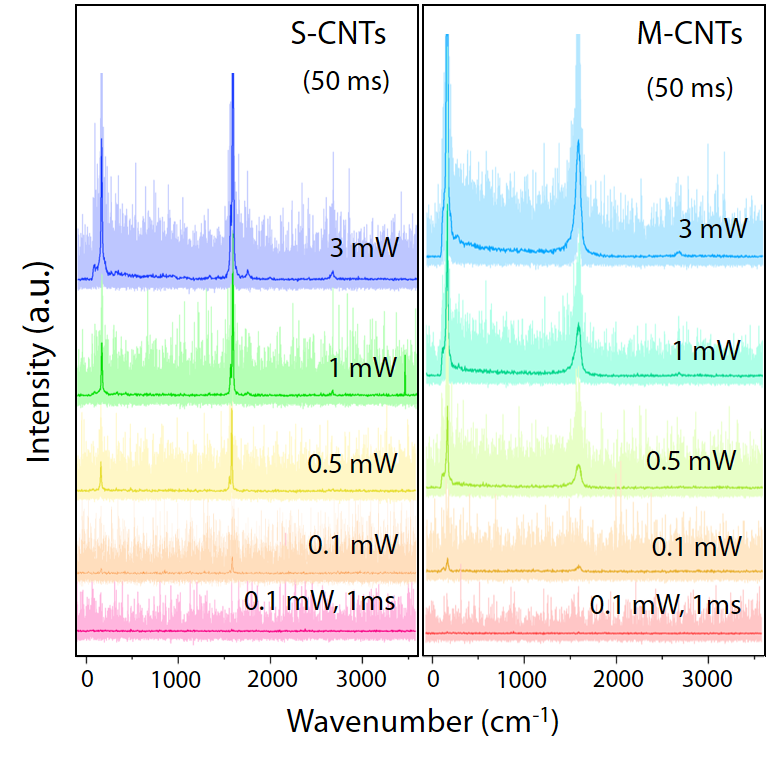


**Fig. S2** Raman spectra of S-CNTs and M-CNTs with various power and a fixed integration time (50 ms). The data are plotted with the same formats as used for Fig. 2a and b.


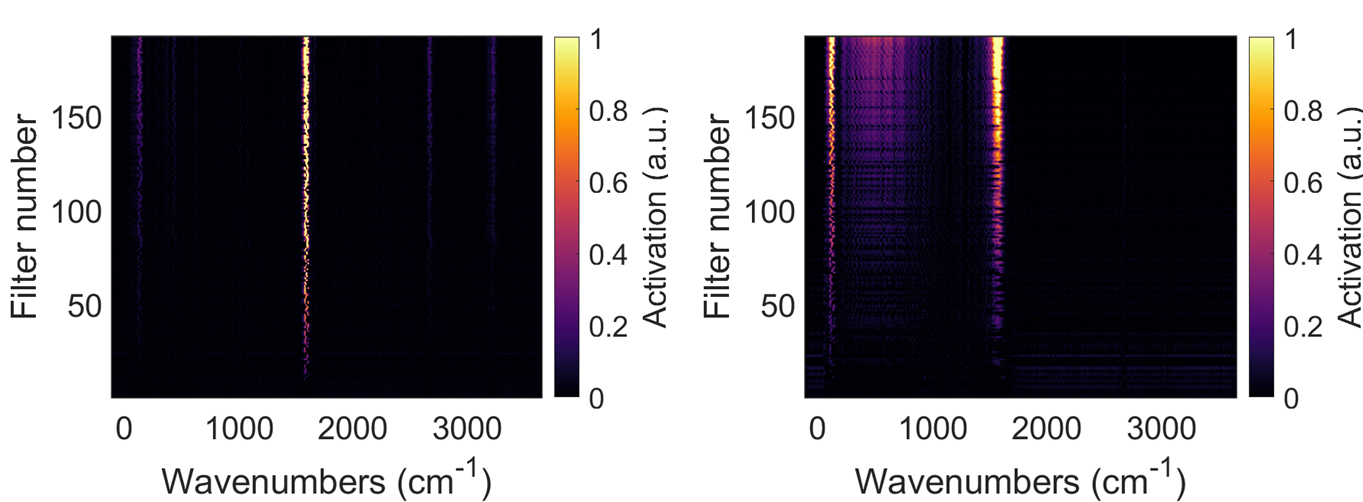


**Fig. S3** Characteristic bands extraction. We plotted the activations values for the first convolutional layer in the plots below for a randomly selected spectrum acquired on a semiconducting (left) and metallic (right) CNT (800ms integration time, 1mW power). The heat maps represent the activation values at the first convolutional layer for all 192 filters. We note that the filters have been sorted according to the maximum activation value. For the S-CNT, the plot shows a sharp peak around ~1600 cm^-1^ (G-peak) and a weak peak around 125 cm^-1^ (RBM). In addition, the 2D peak around 2675 cm^-1^ is also visible. For the M-CNT, also the G-peak is visible around ~1580 cm-1 but slightly broader and asymmetric. In additional, a strong RBM is observed at 135 cm^-1^, together with a broad electronic Raman scat-tering (ERS) signature up to ~600 cm^-1^. This is in agreement with the Raman-active CNT peaks, and corrobo-rates the fact that the network is being activated by the characteristic spectral features.


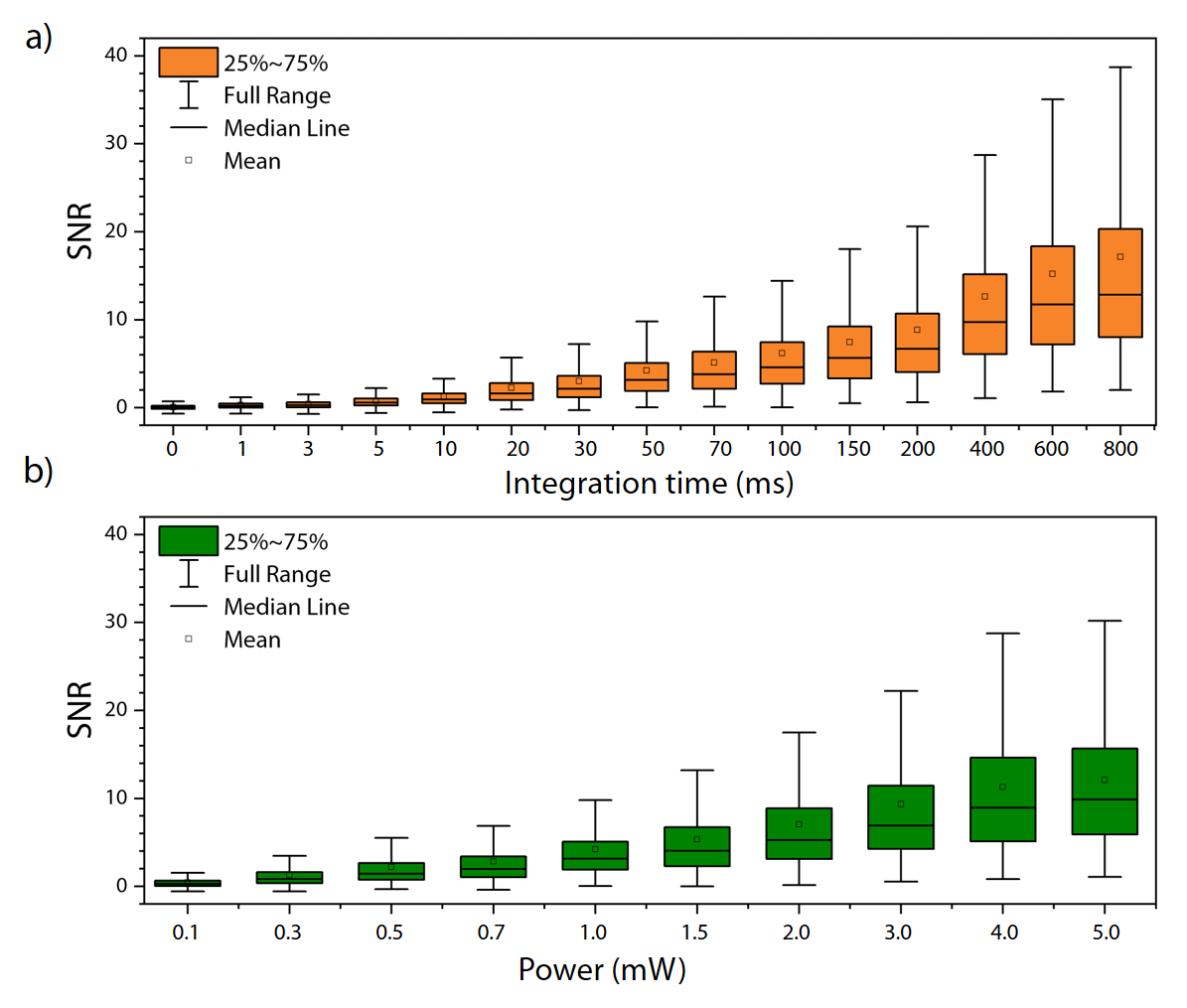


**Fig. S4** SNR plots for all the spectra used for the network training. (a) Box plot of SNR as a function of integration time at a fixed power of 1mW. (b) Box plot of SNR as a function of power at a fixed integration time of 50ms.

**Part 2: Neural network layout and hyperparameter optimization**

The starting layout of the network used in this study is the same ResNet as the one used by Ho *et al^6^.*


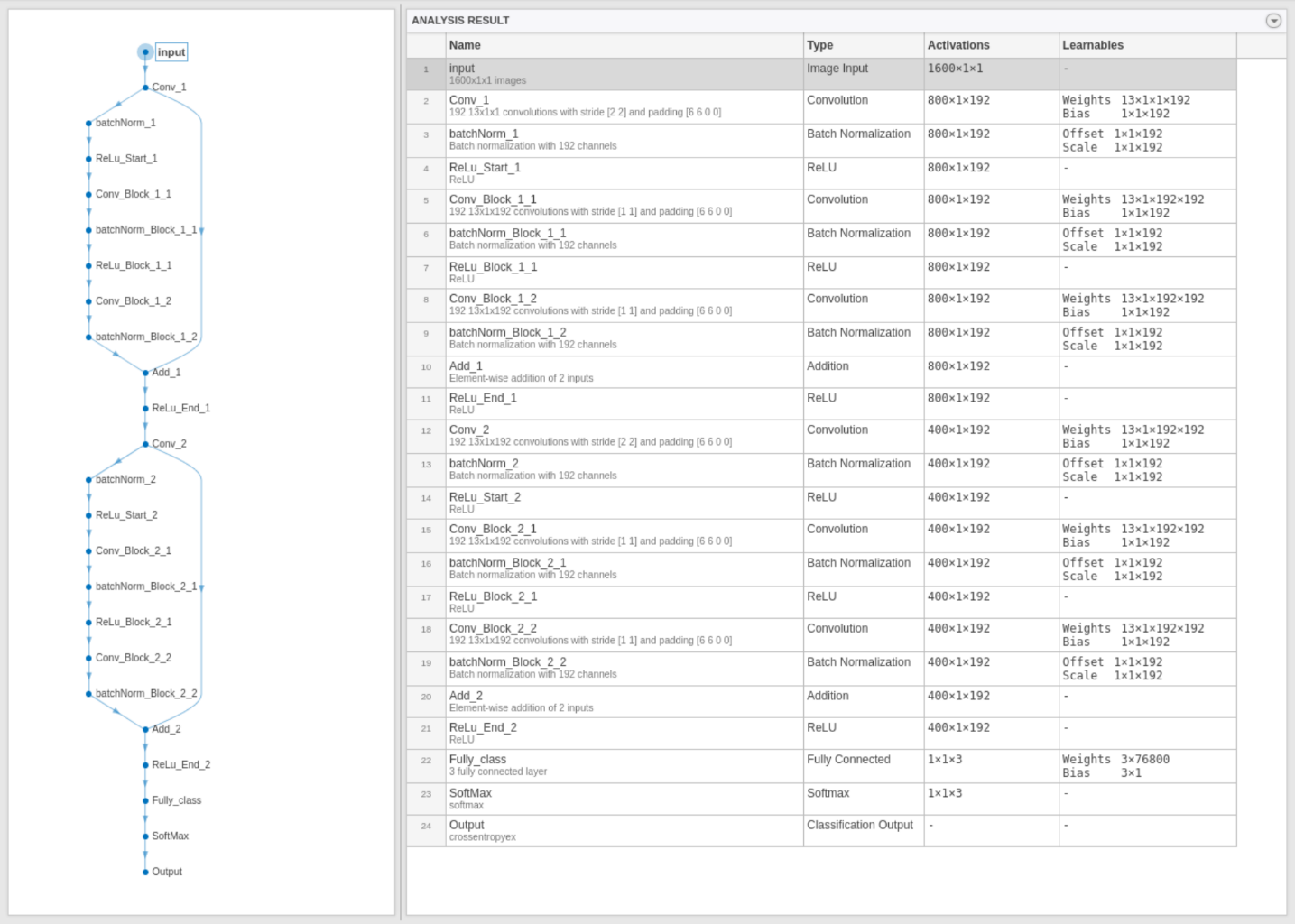
As shown in Fig. S4, the residual network consists of blocks, each block starting with a convolutional layer with a stride of 2, (with batch normalization and a ReLu activation). This initial layer is followed by a set number of convolutional layers with a stride of 1. At the end of each block, before the last ReLu layer, the output of the batch normalization layer is added to the output of the initial convolutional layer (Residual connection). For all convolutional layers, a padding of (filter size - 1) / 2 is used. The residual blocks are followed by a fully connected layer with 3 neurons with a softmax activation function and finally a cross entropy classification layer is utilized.

**Fig. S4** Details of the residual network.

To optimize the network layout, we ran a grid search that systematically varies the number of filters (6x, 32 to 192 in steps of 32), the filter size (6x, 3 to 13 in steps of 2), the number of blocks (6x, 1 to 6 in steps of 1) and the number of convolutional layers per block (5x, 0 to 4 in steps of 1). We note that each block starts with a convolutional layer with stride 2. Here the number of layers refers to the remaining layers in the block with a stride of 1. The number of layers can therefore be zero. As comparison, we also added a regular CNN (without the residual connections) with a stride of 2 for each convolutional layer. This brings the total number of combination to 6^4 = 1296.

For the training of the network, we used a dataset acquired with a power of 1mW and an integration time of 1 ms. The dataset was split in 80%-20% for training/validation, respectively. For each network layout, the mean accuracy of 12 training sessions was used. All networks are trained for 20 epochs with a batch size of 128 and an Adam optimizer with a learning rate of 1e^-3^.

For the analysis of the results, we first selected the best 20 performing network layouts. For these selected networks, we plot (in blue lines) the dependence of the accuracy on the 4 hyperparamters that have been optimized (see Fig. S5). In the figure, we also present in the background a density plot of all 216 lines (6^3).

The first observation (a) is that more blocks in the network yield a lowering in accuracy, with a maximum at 1 layer. The same plot also shows that the regular CNN performs better than the ResNet architecture on this dataset. The figure (b) shows that the number of blocks has a maximum at 4 for the best performing network, while in general a slight downward trend is observed for an increasing number of block. For the filter size (c), both the lines and the density plot exhibit in increase in accuracy with filter size. A similar observation can be made for the number of filters (d), the more the better.


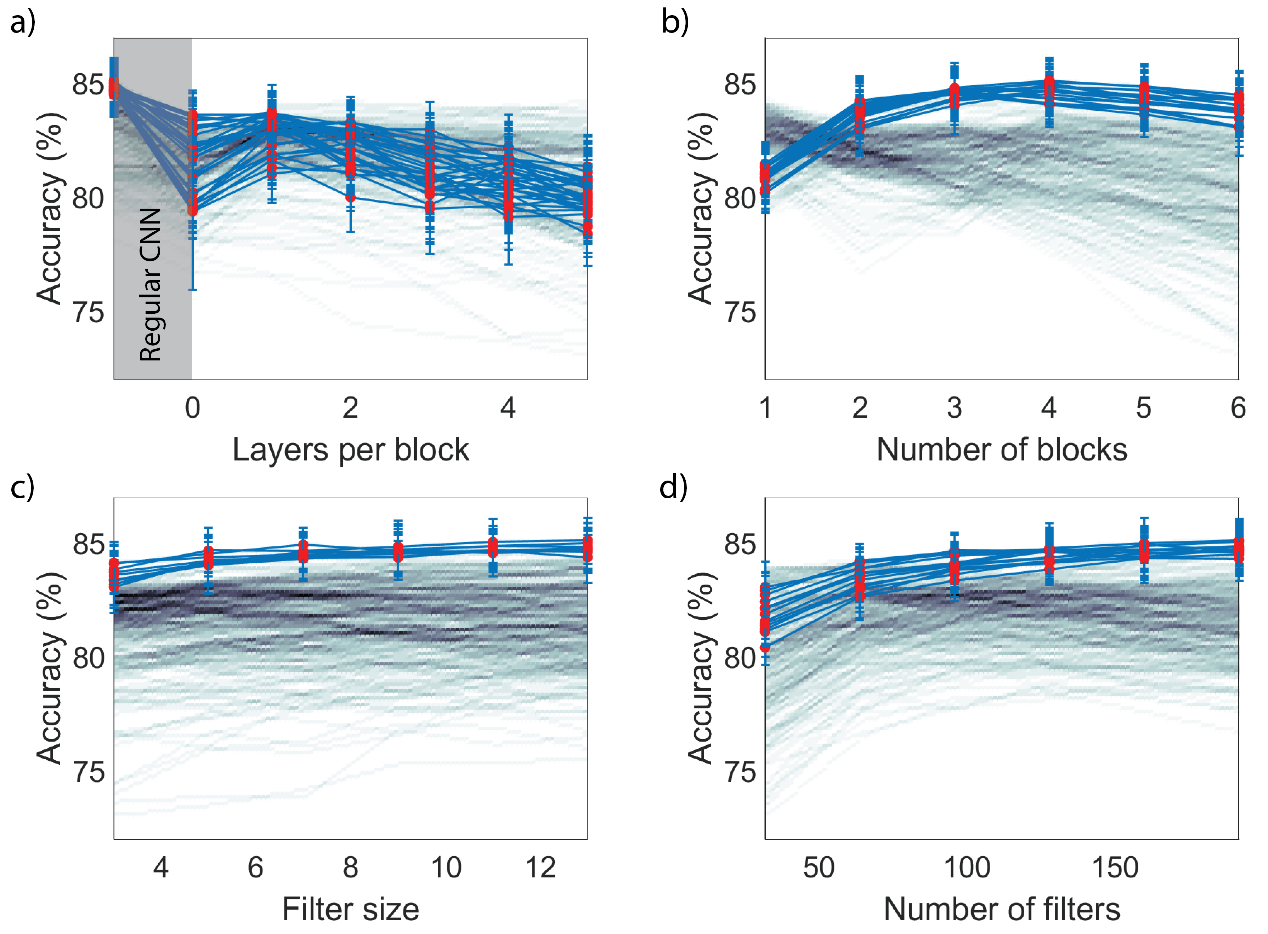


**Fig. S5** Dependence of the accuracy on 4 hyperparamters

Based on these observations, we conclude the best performing network is a regular CNN with 4 convolutional layers, with 192 filters of size 13, as shown in Fig. S6.


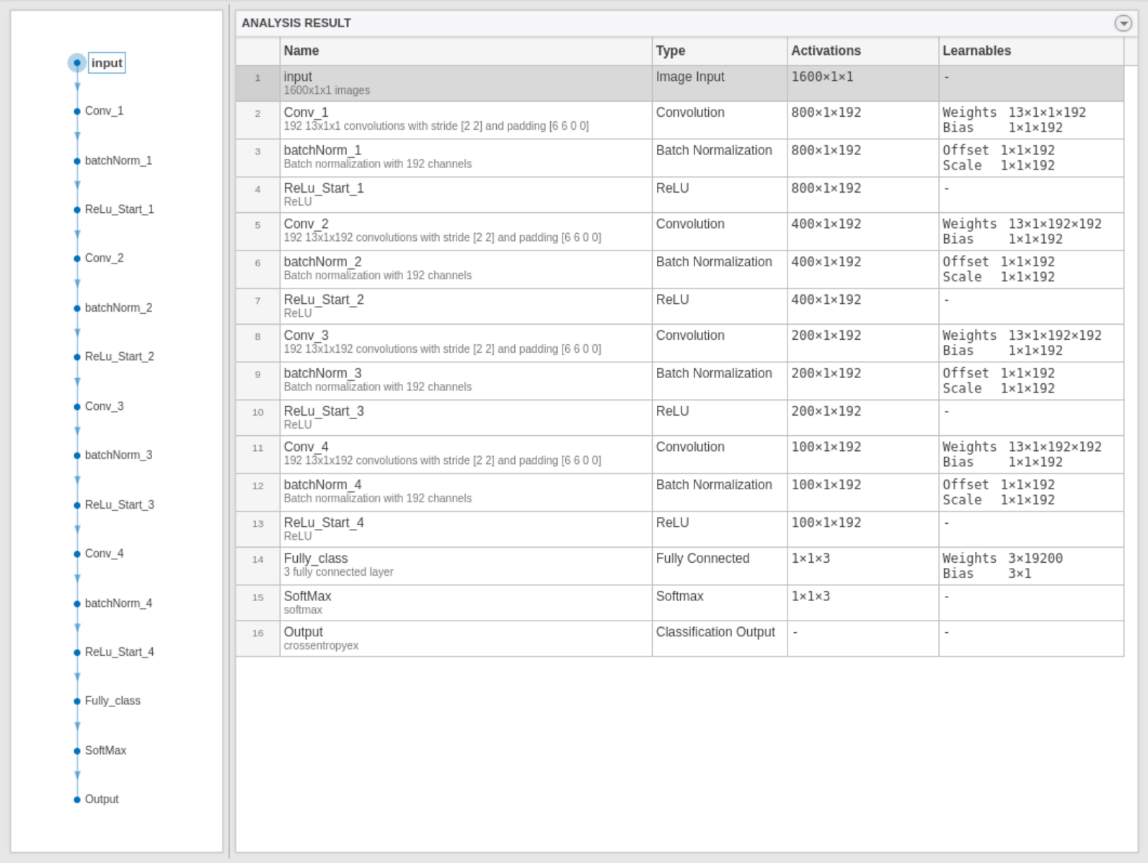


**Fig. S6** The best performing network with 4 convolutional layers, with 192 filters of size 13.

**REFERENCES**

1. Dresselhaus, M. S., Dresselhaus, G., Saito, R. & Jorio, A., Raman spectroscopy of carbon nanotubes. *Phys. Rep.* **409**, 47-99 (2005).
2. Dresselhaus, M. S., Jorio, A. & Saito, R., Characterizing graphene, graphite, and carbon nanotubes by Raman spectroscopy. *Annu. Rev. Condens. Matter Phys.* **1**, 89-108 (2010).
3. Farhat, H. et al. Observation of electronic Raman scattering in metallic carbon nanotubes. *Phys. Rev. Lett.* **107**, 157401 (2011).
4. Zhang, D., Yang, J., Li, M. & Li, Y. (n, m) Assignments of Metallic Single-Walled Carbon Nanotubes by Raman Spectroscopy: The Importance of Electronic Raman Scattering. *ACS Nano* **10**, 10789-10797 (2016).
5. Jung, S., Hauert, R., Haluska, M., Roman, C. & Hierold, C. Understanding and improving carbon nanotube-electrode contact in bottom-contacted nanotube gas sensors. *Sensor Actuat. B-Chem.* **331**, 129406 (2021).
6. Ho, C. S. et al. Rapid identification of pathogenic bacteria using Raman spectroscopy and deep learning. *Nat. Commun.* **10**, 4927 (2019).
